# Immunohistochemical and in situ hybridization study of urate transporters GLUT9/URATv1, ABCG2, and URAT1 in the murine brain

**DOI:** 10.1186/s12987-016-0046-x

**Published:** 2016-12-12

**Authors:** Naoko H. Tomioka, Yoshifuru Tamura, Tappei Takada, Shigeru Shibata, Hiroshi Suzuki, Shunya Uchida, Makoto Hosoyamada

**Affiliations:** 1 Department of Human Physiology and Pathology, Faculty of Pharma-Sciences, Teikyo University, 2-11-1 Kaga, Itabashi-ku, Tokyo, 173-8605 Japan; 2 Department of Internal Medicine, Teikyo University School of Medicine, Teikyo University, 2-11-1 Kaga, Itabashi-ku, Tokyo, 173-8605 Japan; 3 Department of Pharmacy, The University of Tokyo Hospital, Faculty of Medicine, The University of Tokyo, 7-3-1 Hongo, Bunkyo-ku, Tokyo, 113-8655 Japan

## Abstract

**Background:**

Uric acid (UA) is known to exert neuroprotective effects in the brain. However, the mechanism of UA regulation in the brain is not well characterized. In our previous study, we described that the mouse urate transporter URAT1 is localized to the cilia and apical surface of ventricular ependymal cells. To further strengthen the hypothesis that UA is transported transcellularly at the ependymal cells, we aimed to assess the distribution of other UA transporters in the murine brain.

**Methods:**

Immunostaining and highly-sensitive in situ hybridization was used to assess the distribution of UA transporters: GLUT9/URATv1, ABCG2, and URAT1.

**Results:**

Immunostaining for GLUT9 was observed in ependymal cells, neurons, and brain capillaries. Immunostaining for ABCG2 was observed in the choroid plexus epithelium and brain capillaries, but not in ependymal cells. These results were validated by in situ hybridization.

**Conclusions:**

We propose that given their specific expression patterns in ependymal, choroid plexus epithelial, and brain capillary endothelial cells in this study, UA may be transported by these UA transporters in the murine brain. This may provide a novel strategy for targeted neuroprotection.

## Background

Uric acid (UA) exerts a neuroprotective effect due to its antioxidant property, and epidemiological and experimental evidence suggests that UA plays an important role in the development or progression of neurodegenerative disorders [1]. For instance, higher serum UA is associated with the decreased incidence and slower progression of Parkinson’s disease (PD) [2]. Moreover, recent studies indicate that UA transporter genes, which control the transport of UA in the kidney and extra renal tissues, and thus affect serum UA levels, are also associated with the risk and age at onset of PD [3–6]. In rodent models of PD, elevated UA levels attenuated behavioral and neurodegenerative deficits [7, 8]. Urate-elevating clinical trials are currently underway in patients with the early stages of PD. An oral administration of inosine, a precursor of UA, can elevate UA levels in serum and cerebrospinal fluid (CSF), with a persistent elevation of plasma antioxidant capacity [9, 10]. Further, CSF UA is inversely correlated with the clinical progression of PD, albeit to a lesser extent than serum UA [11]. However, the molecular mechanism as to how the UA in blood reaches the brain parenchyma and affects neuronal viability remains unclear.

We previously demonstrated that URAT1, which is a urate transporter responsible for urate reabsorption in the kidney [12], is localized to cilia and the apical surface of ventricular ependymal cells in the murine brain [13]. Ependymal cells form a single-layer of epithelial cells which line the surface of the cerebral ventricles. Although the lateral ventricular CSF-brain interface does not usually act as a barrier due to the lack of tight junctions and may allow passive molecular exchange, immunoreactivity of tight junction proteins has been demonstrated in the ependymal cells of specific regions of the third and fourth ventricles [14–17]. Therefore, alternative carrier-mediated transport systems may exist at the ependymal layer in addition to slow paracellular diffusion. For example, a recent study indicates that the glutamate transporter EAAT1, which is localized on the apical membrane of the ependymal cell is involved in the removal of l-Glutamate from the CSF [18]. It is also known that proximal tubules which express functional UA transporters, are leaky epithelial cells [19]. In this regard, we hypothesized that ependymal URAT1 and other transporters may function as a UA transporter between the ventricular CSF and the interstitial fluid of the brain parenchyma.

To further strengthen the hypothesis that UA transport systems exist in ependymal cells, the aim of this study was to address if other UA transporters were also localized in those cells. In this study, we focused on two other UA transporters, GLUT9/URATv1 and ABCG2, which are known to regulate serum UA levels [20]. RT-PCR analyses showed that mRNA encoding the long isoform of GLUT9 is found both in the human and murine brain [21, 22]. Furthermore, GLUT9 is also expressed in cultured dopaminergic neurones and astroglial cells [23]. However, the spatial distribution of GLUT9 in the brain is still unknown. Further, while ABCG2 luminal localization in brain capillaries, and on murine choroid plexus epithelial cells has been previously described [24, 25], its localization on ependymal cells is still unknown.

Thus, the aim of this study was to investigate the distribution of GLUT9 and ABCG2 in the murine brain, particularly in ependymal cells. To do this, we performed immunostaining and highly-sensitive in situ hybridization analyses of the murine brain.

## Methods

### Animals

A total of seven male C57BL/6J mice (Sankyo Laboratories, Tokyo, Japan), a male Abcg2-knockout (KO) mouse (FVB.129P2-Abcg2, Taconic Farms, Hudson, NY), and a littermate wild-type (WT) mouse were used in this study. Mice were maintained in 12 h light and dark cycles, with free access to food and water. All animal experiments were carried out in accordance with the guidelines for animal experimentation in Teikyo University and the University of Tokyo, and the project was approved by the local committee.

### Tissue section preparation

To prepare fixed, frozen sections, mice were anesthetized by pentobarbital injection (50 mg/kg, i.p.) and perfused intracardially with HEPES buffer (30 mM HEPES, 100 mM NaCl, 2 mM CaCl_2_, pH 7.4), followed by 4% paraformaldehyde (PFA) in HEPES buffer. Brains were then removed and post-fixed for 3 h at 4 °C in the same fixative. The post-fixed brains were cut coronally and cryoprotected in 15% sucrose (wt/vol) in PBS for 48 h at 4 °C, embedded in Tissue-Tek OCT compound (Sakura Finetek Japan, Tokyo, Japan), and frozen on dry ice. Sections were cut at 12 μm for immunostaining.

To prepare paraffin sections, anesthetized mice were perfused intracardially with methacarn fixative (methanol:chloroform:acetic acid = 6:3:1). Brains were removed, cut coronally, and post-fixed 2 h at 4 °C in the same fixative. For paraffin embedding, tissues were dehydrated in a graded series of alcohols, cleared with Hemo-De (a Xylene substitutive, FALMA, Tokyo, Japan), embedded in Paraplast Plus (Sigma, St. Louis, MO, USA), and sectioned at 4 μm.

To prepare unfixed, fresh frozen sections for immunostaining, mice were killed by cervical dislocation and the brains were removed and frozen on dry ice. Sections were cut at 20 μm on a cryostat. To prepare paraffin sections for in situ hybridization, the anesthetized mouse was perfused intracardially with PBS, followed by 4% PFA in PBS. Brains were removed and cut coronally, post-fixed overnight (i.e. for at least 16 h), at 4 °C in 4% PFA in PBT (PBS containing 0.1% Tween 20). After rinsing in PBT, tissues were dehydrated in a graded series of ethanol in PBT, cleared with Hemo-De, embedded in Paraplast Plus and sectioned at 5 μm.

### Immunostaining for brain sections

For immunofluorescence staining of cryostat sections, sections were autoclaved in 10 mM citrate buffer, pH 6.0, for 5 min at 105 °C for antigen retrieval, and incubated in blocking solution (PBS containing 10% normal goat serum and 0.1% Triton X-100) for 1 h at room temperature (22–25 °C). The sections were then subsequently incubated with primary antibodies overnight at 4 °C and with Alexa 488- or Alexa 594-conjugated secondary antibodies (Molecular Probes, Tokyo, Japan) for 1 h at room temperature. Slides were mounted with Vectashield containing DAPI (Vector Laboratories, Burlingame, CA, USA).

Paraffin sections were deparaffinized with Hemo-De and rehydrated prior to staining. For immunohistochemistry, sections were further incubated in 0.3% H_2_O_2_/methanol solution for 10 min to block the endogenous peroxidase activity. Sections were then incubated for 1 h at room temperature in blocking solution, which consisted of 5% normal goat serum and 0.1% Triton X-100, followed by an overnight-incubation at 4 °C with primary antibodies diluted in blocking solution. Detection was performed with EnVision™ + Systems and DAB+, Liquid (Dako Japan, Tokyo, Japan). Sections were counterstained with Mayer’s hematoxylin (Wako, Tokyo, Japan). Immunofluorescence staining of the rehydrated sections was performed using the protocol outlined above for cryostat sections, but without the antigen retrieval. For immunofluorescence staining of fresh frozen sections, sections was fixed in methanol (−20 °C, 30 min) and acetone (4 °C, 10 min), incubated with blocking buffer, primary, and fluorescently-labelled secondary antibodies. The following primary antibodies were used in this study: anti-GLUT9 (NBP1-05054, Novus Biologicals, 1/500 or 1/1000 dilution for immunofluorescence staining, 1/5000 dilution for immunohistochemistry); anti-acetylated-α-tubulin (6-11B-1, Sigma, 1:500); anti-NeuN (A60, Millipore, 1/500); anti-P Glycoprotein (C219, GeneTax, 1/200); anti-BCRP/ABCG2 (BXP-9, Abcam, 1/100). For antigen absorption experiment, GLUT9 peptide (NBP1-05054PEP, Novus Biologicals) was used at a concentration of 100 μg/ml.

### In situ hybridization

In situ hybridization was performed using ViewRNA™ ISH Tissue 2-Plex assay (Affymetrix, Tokyo, Japan) according to the manufacturer’s protocol. Paraffin sections were deparaffinized with Hemo-De, boiled in pretreatment solution for 10 min, and digested with protease for 10 min. Sections were then hybridized with ViewRNA probes (Mouse Slc22a12, Type 6; Mouse Slc2a9, Type 1: Mouse Abcg2, Type 1). Bound probes were amplified by hybridization with preamplifier and amplifier mix solutions. Sections were incubated with Label Probe 6-alkaline phosphatase (AP) solution and Fast Blue substrate. Subsequently, sections were incubated with Label Probe 1-AP Type 1 solution and Fast Red substrate. Finally, the sections were counterstained with Gill’s hematoxylin and cover-slipped with Ultramount Permanent Mounting Medium (Dako Japan, Tokyo, Japan).

### Image acquisition

DAB-stained sections were scanned with a NanoZoomer 2.0-HT slide scanner (Hamamatsu Photonics, Hamamatsu, Japan). For immunofluorescence experiments, single-plane images were captured using a Nikon A1 confocal microscope (Nikon, Tokyo, Japan) with identical settings. In situ hybridization, fluorescence, and bright field images were also acquired with the Nikon A1 confocal microscope. The Fast Blue and Fast Red signals were observed using Alexa 750 and Cy3 filter sets, respectively.

## Results

### Immunofluorescence staining of GLUT9 in PFA-fixed murine brain sections

**Fig. 1:**
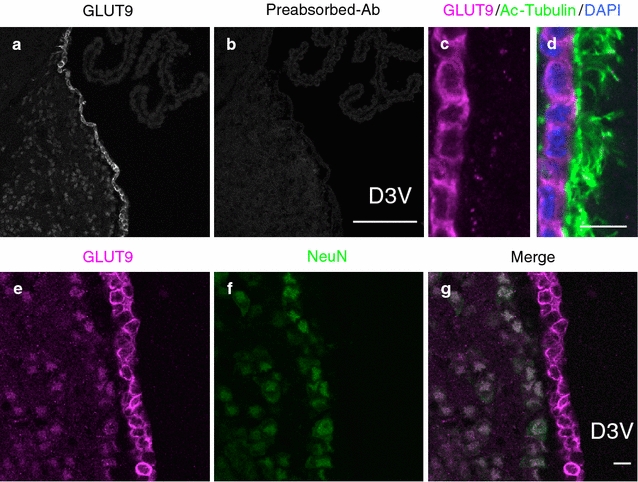
Immunofluorescence staining of GLUT9 in PFA-fixed murine brain sections. Frozen sections of paraformaldehyde-fixed wild-type murine brain were used for immunofluorescence staining. **a**, **b** Antigen absorption test. Immunofluorescence staining of the ependymal wall of the dorsal third ventricle using **a** anti-GLUT9 antibody and **b** antigen-preabsorbed antibody. *Scale bar* 100 µm. **c**, **d** Immunofluorescence staining of GLUT9 (*magenta*), acetylated-tubulin (Ac-Tubulin, *green*) and DAPI (*blue*) on ependymal cells. *Scale bar* 10 µm. **e**–**g** Immunofluorescence staining of GLUT9 (*magenta*) and NeuN (*green*) showing co-localization in neurons. *Scale bar* 10 µm. D3V, dorsal third ventricle; DAPI, 4′,6-diamidino-2-phenylindole; NeuN, neuronal nucleus marker

To determine whether GLUT9 is localized in ependymal cells, we first performed immunostaining analysis using a rabbit polyclonal anti-GLUT9 antibody on PFA-fixed murine brain frozen sections. Distinct GLUT9 immunoreactivity in the ependymal cells of the dorsal third ventricle, and weaker staining in the brain parenchyma region were detected (Fig. 1a). The specificity of anti-GLUT9 antibody was verified by antigen absorption test (Fig. 1b). Higher magnification imaging revealed that GLUT9 was not localized to the nucleus or the cilia, visualized using anti-acetylated-α-tubulin, suggesting an intracellular and plasma membrane localization (Fig. 1c, d). To identify the cell type of GLUT9-positive cells in the brain parenchyma, we performed double immunostaining for GLUT9 and NeuN (neuronal marker; Fig. 1e–g). All GLUT9-positive cells in the parenchyma co-localized with NeuN, suggesting that GLUT9 is also present in neurons.

### GLUT9 immunoreactivity is detected in ependymal cells of all ventricles

**Fig. 2:**
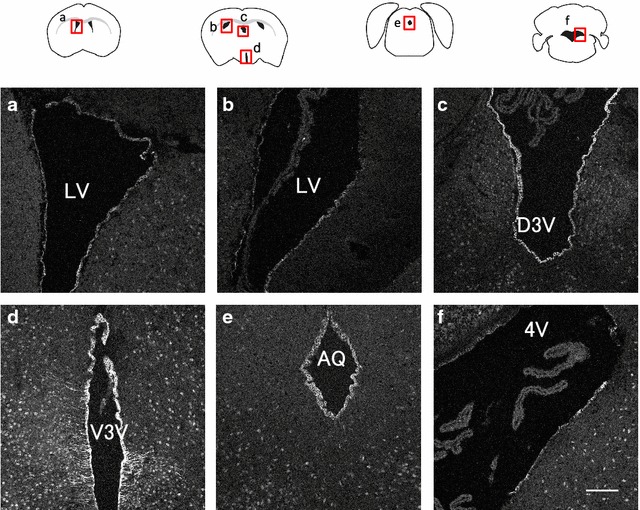
GLUT9/URATv1 immunoreactivity is detected in ependymal cells of all ventricles. Frozen sections of paraformaldehyde-fixed wild-type murine brain were used for immunofluorescence staining. The *red squares* in the diagrams indicate the region shown in each image. **a**–**f** GLUT9 staining of coronal sections. **b**–**d** Images were obtained from the same section. *Scale bar* 100 µm. LV, lateral ventricle; V3V, ventral third ventricle; AQ, aqueduct; 4V, fourth ventricle

Next, we observed the distribution of GLUT9 in other ventricles using PFA-fixed, frozen coronal sections at different levels. In addition to the ependymal cells of dorsal third ventricle (Figs. 1a, 2c), GLUT9 immunoreactivity was detected in ependymal cells of all ventricles including the lateral, ventral third, and fourth ventricles, and the aqueduct (Fig. 2a, b, d–f). GLUT9 was also prominent in tanycytes, which are specialized ependymal cells that line the floor of the ventral third ventricle, and in its long processes that extend into the parenchyma (Fig. 2d). No immunostaining was detected in the choroid plexus (Fig. 2b, c, f).

### Immunohistochemistry and immunofluorescence staining of GLUT9 in a methacarn-fixed murine brain

**Fig. 3:**
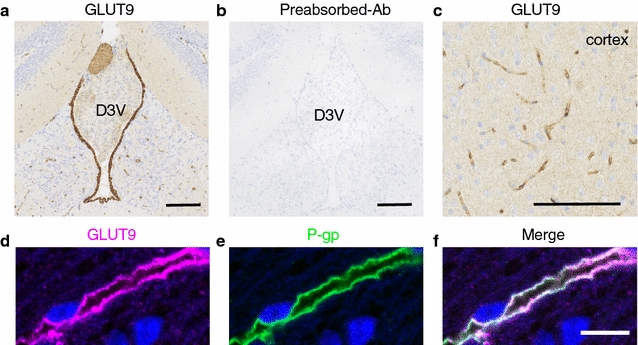
Immunohistochemistry and immunofluorescence staining of GLUT9 in methacarn-fixed murine brain. Paraffin sections of methacarn-fixed murine brain were used for immunostaining. **a**, **b** Antigen absorption test. Immunohistochemistry using showing **a** anti-GLUT9 antibody staining in the third ventricle ependyma and parenchyma and **b** antigen-preabsorbed antibody. *Scale bar* 100 µm. **c** GLUT9 immunoreactivity observed in brain capillaries in the cortex. *Scale bar* 100 µm. **d**–**f** Immunofluorescence staining of **d** GLUT9 and **e** P-gp showing co-localization in capillaries. *Blue* indicates DAPI-stained nucleus of the brain capillary endothelial cell. *Scale bar* 10 µm. P-gp, P-glycoprotein

Next, we performed immunostaining analysis using methacarn-fixed tissue. Similar to the results obtained from PFA-fixed tissue, GLUT9 immunoreactivity was detected in ependymal cells (Fig. 3a). In addition, capillary-like structures were also immunopositive for GLUT9 in the brain parenchyma, including the cortical region (Fig. 3a, c). No staining was detected with antigen-preabsorbed antibody (Fig. 3b). To investigate the distribution of GLUT9 in the brain capillary endothelium, we conducted double-immunostaining with anti-GLUT9 and anti-P-glycoprotein (P-gp) antibody, which is a luminal marker (Fig. 3d–f). GLUT9 co-localized with P-gp (Fig. 3f), indicating that GLUT9 possibly localizes to the luminal membrane of the brain capillary endothelium.

### Immunofluorescence staining of ABCG2 in methanol/acetone-fixed and methacarn-fixed murine brain

**Fig. 4:**
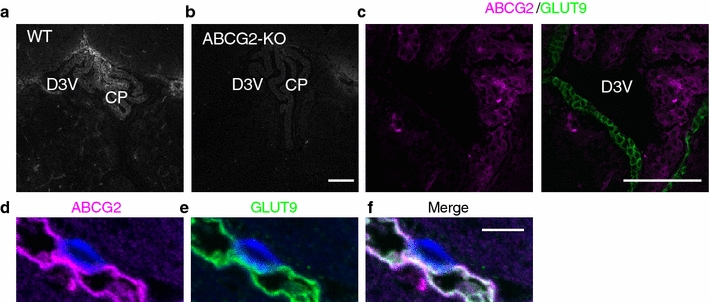
Immunofluorescence staining of ABCG2 in methanol/acetone-fixed and methacarn-fixed murine brain. Fresh frozen sections of the dorsal third ventricle were prepared from wild-type (WT) and ABCG2 knockout (KO) mice and post-fixed with methanol and acetone. Immunofluorescence staining of ABCG2 was seen in choroid plexus and capillaries in sections from **a** WT but not in **b** ABCG2 KO mouse. *Scale bar* 100 µm. **c** Immunofluorescence staining of ABCG2 (*magenta*) and GLUT9 (*green*) showing ABCG2 in the choroid plexus and GLUT9 in the ependyma. *Scale bar* 100 µm. **d**–**f** Immunofluorescence staining of **d** ABCG2 and **e** GLUT9 using methacarn-fixed paraffin section of capillaries showing co-localization in **f**. *Blue* indicates DAPI-stained nucleus of the brain capillary endothelial cell. *Scale bar* 10 µm. CP, choroid plexus; D3V, dorsal third ventricle

Immunohistochemistry of ABCG2 was done on fresh, frozen sections of the wild type (Fig. 4a) and Abcg2 KO (Fig. 4b) mice, which were post-fixed with methanol and acetone. ABCG2 immunoreactivity on the luminal membrane of the capillary endothelium and the CSF side of the choroid plexus epithelial cells has been previously reported [25]. Using a different antibody, we also demonstrated a similar distribution of ABCG2 in the capillary endothelium and choroid plexus epithelial cells (Fig. 4a). These immunoreactivity patterns were not observed in sections from the Abcg2 KO mouse (Fig. 4b), demonstrating antibody specificity. ABCG2 immunoreactivity was not detected in ependymal cells (Fig. 4c). Using paraffin sections from methacarn-fixed brain, we observed the colocalization of ABCG2 and GLUT9 on the capillary endothelium (Fig. 4d–f).

### Localization of mRNA of urate transporters in the murine brain by fluorescence in situ hybridization

**Fig. 5:**
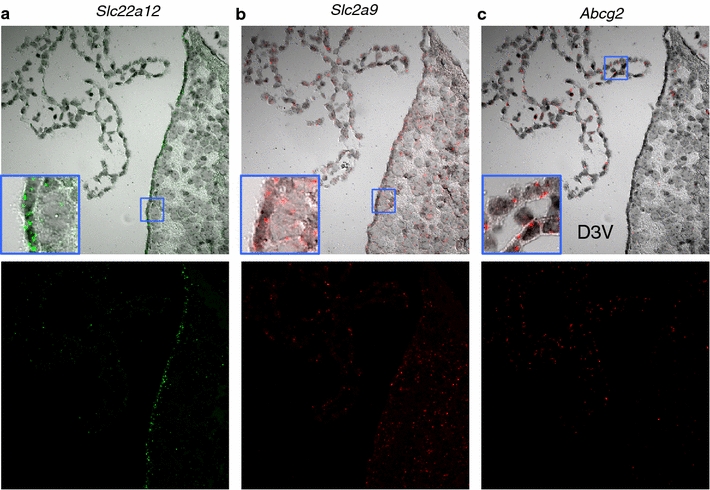
Localization of mRNA of urate transporters in murine brain by fluorescence in situ hybridization. **a**–**c** Merged bright field and fluorescence images are seen in the *upper panels*, while the *lower panels* show the fluorescence images alone. *Green dots* show Fast Blue signal observed with the Alexa 750 filter set, while the red dots show Fast Red signal observed with the Cy3 filter set. mRNA probes: Slc22a12 (URAT1), Slc2a9 (GLUT9), Abcg2 (ABCG2). Slc22a12 is expressed largely in the ependyma and less in the parenchyma, Slc2a9 is expressed in the ependyma and parenchyma and Abcg2 in the choroid plexus epithelium

The distribution of urate transporters in the murine brain was further verified by a highly-sensitive in situ hybridization system using *Slc22a12* (URAT1), *Slc2a9* (GLUT9), and *Abcg2* probes. In accordance with our previous URAT1 immunostaining results, where URAT1 was distributed in ependymal cells [13], *Slc22a12* mRNA was expressed in the ependymal cells (Fig. 5a). Weaker signals were observed in choroid plexus and brain parenchyma where the protein localization was not confirmed (Fig. 5a). *Slc2a9* mRNA was expressed broadly in ependymal cells, the choroid plexus, and brain parenchyma (Fig. 5b). *Abcg2* mRNA was expressed in choroid plexus epithelial cells and weakly in brain parenchyma, but not in ependymal cells (Fig. 5c). These results establish the validity of immunostaining results, which revealed the distribution of URAT1 and GLUT9 in ependymal cells, and of ABCG2 in the choroid plexus.

## Discussion

In the current study, we showed that GLUT9 is expressed in ependymal cells, neurons, and brain capillaries, while ABCG2 is expressed in choroid plexus epithelium and brain capillaries, but not in ependymal cells. Taken together with our previous findings that URAT1 is localized at the CSF side of the ependymal cells, we speculate that UA may be transported via transporters expressed at the cells which form the boundary between brain, CSF and blood.

While the immunoreactivity of GLUT9 in the ependymal cells was consistently observed in our experiments, its immunoreactivity in neurons or brain capillaries was dependent on fixation or antigen retrieval conditions. The difference in the immunostaining pattern may be caused by antigen masking or degradation. The preservation of antigenicity can be affected by fixation methods and may vary among tissues. Methacarn fixation is a non-cross-linking organic solvent, which has been shown to improve immunoreactivity, in comparison to aldehyde-based fixatives, against particular antigens [26]. The neuronal expression of GLUT9 is feasible, since its expression in cultured dopaminergic neurons has been previously demonstrated using western blot [23]. Two isoforms of GLUT9, which differ in the amino terminus, are known to exist in the human and mouse. The long isoform of human GLUT9 is expressed at the basolateral membrane of proximal tubules of human kidney, whereas the short isoform is expressed at the apical membrane of the collecting duct [21, 27]. In contrast, mouse GLUT9 is reportedly expressed both in the apical and basolateral membranes of distal convoluted tubules of the murine kidney and enterocytes of the murine jejunum, albeit with no information about its isoform-specific localization [22, 28, 29]. Since the current study did not reveal the exclusive plasma membrane localization compared to our previous finding of apical localization of URAT1 [13], further work including electron microscopy analysis is required to determine if GLUT9 is specifically localized in the apical and/or basolateral membrane of ependymal cells and neuronal somatic membranes. Unknown mechanisms, such as a stimulus-dependent translocation to the plasma membrane like the insulin-dependent GLUT4 translocation may exist [30]. Surprisingly, we found that methacarn fixation alone showed the localization of GLUT9 on brain capillary endothelial cells. This result supports RT-PCR findings that GLUT9 mRNA is detected in bEnd.3 cells (a murine brain endothelial cell line) [31, 32].

The protein expression of URAT1 and GLUT9, but not ABCG2, in the ependymal cells was also validated by the use of highly-sensitive in situ hybridization. Similarly, the expression of ABCG2 in choroid plexus cells was also confirmed. *Slc2a9* and *Abcg2* mRNA distribution in the brain parenchyma may correspond to the protein localization in neurons and brain capillary endothelial cells. Conversely, *Slc22a12* mRNA signals were observed in the choroid plexus and the brain parenchyma, and *Slc2a9* mRNA signals were observed in the choroid plexus, which is inconsistent with the immunostaining results. Immunoblotting analysis in earlier studies has reported that URAT1 was detected in the murine choroid plexus, brain capillaries, cultured dopaminergic neurons, and astrocytes [23, 33]. Our previous study exhibited weak immunostaining in the choroid plexus and the brain parenchyma, both in wild type and URAT1 KO mice, indicating the possibility of non-specific staining [13]. The discrepancy between the immunostaining and in situ hybridization data of GLUT9 (*Slc2a9*) and URAT1 (*Slc22a12*) could be considered that the mRNA is not translated to protein or that the translated protein is unstable. Alternatively, other optimum fixative conditions for immunostaining may be needed.

Specific localization of UA transporters in the murine brain could be involved in the transport of UA derived from blood or by intracellular purine metabolism. Considering the correlation between blood and CSF UA levels [34], we suppose that brain interstitial UA could be mainly provided by CSF, which originates from blood at the choroid plexus. The endothelial cells of the choroid plexus are fenestrated and tight junctions of choroid plexus epithelial cells constitute the blood-CSF barrier [35]. Our group and others have demonstrated that ABCG2 is localized at the apical membrane of choroid plexus epithelial cells [25], raising the possibility that UA may be partly secreted by ABCG2 into the ventricular CSF. An unidentified UA transporter at the blood side may be involved in the uptake of blood UA into the choroid plexus epithelial cells. CSF flows through the ventricles, enters into the subarachnoid space, and is reabsorbed into the blood or reaches lymphatic drainage [36]. During the ventricular circulation, UA in the CSF could be transported into ependymal cells by URAT1 located at the apical membrane of ependymal cells [13]. Since it has been reported that ependymal cells express xanthine oxidase, which converts hypoxanthine to xanthine and xanthine to uric acid [37], metabolized UA in the ependymal cells could also be the transport target. If GLUT9 is localized at the basolateral membrane, then it is likely to contribute to the transport of UA from ependymal cells to brain parenchyma. CSF-derived UA could increase the regional UA concentration near the ventricle, for example in the striatum near the lateral ventricles. Conversely, apically-localized GLUT9 could transport UA from ependymal cells to the ventricular CSF. It has been demonstrated that ATP-binding cassette transporters ABCG2 and MRP4/ABCC4 that transport UA are localized to luminal membrane of endothelial cells [20, 25, 38]. At the abluminal membrane, OAT3, which can function as a urate/dicarboxylate exchanger, is present [39, 40]. In addition to these transporters, we revealed that GLUT9 also exists at the capillary endothelial cells, albeit its luminal/abluminal distribution should be further investigated by electron microscopy analysis. We postulate that at the blood–brain barrier, UA in the capillary endothelial cell is excreted into blood via luminal ABCG2 and GLUT9 may also be involved in the UA transport at this site.

In the present paper, we have mainly discussed about the possible role of brain GLUT9 and ABCG2 regarding the transport activity of UA [41–43]. However, association with other candidate substrates of the transporters needs to be taken into consideration. Initially, GLUT9 was shown to be a high-affinity, low-capacity glucose and fructose transporter [44]. Other GLUT transporters are also expressed in the brain [45] and among them, GLUT1 is highly expressed in both the luminal and abluminal membranes of the endothelial cells and predominantly involved in the glucose transport into the brain [46, 47]. Whether the brain UA transporters are actually involved in the UA transport at the ependymal cells, choroid plexus epithelial cells and brain capillary endothelial cells and the effect of competitive substrates should be ascertained in future studies including the direct measurement of local UA at the cellular level.

It has recently been reported that genetic variants of *SLC2A9* (GLUT9) and *ABCG2*, which influence the serum level of UA, can modify susceptibility to PD [3, 5, 6]. Further, an elevated/reduced UA level in serum correlates closely with an elevated/reduced UA level in the brain [34]. Moreover, the dysfunction of regional UA transport in the brain may have additional effects on the level of UA in the brain. GLUT9 dysfunction in the ependymal cells may reduce the UA supply for brain parenchyma. ABCG2 dysfunction in the brain capillary endothelial cells may inhibit UA excretion into blood and maintain the brain parenchyma UA level. Since multiple UA transporters exist on the luminal side of endothelial cells, further investigation is required to reveal the main driver of UA transport.

## Conclusions

In this study, we propose that UA in the brain could be transported by UA transporters, which show specific expression patterns in ependymal, choroid plexus epithelial and brain capillary endothelial cells. Thus far, the importance of UA in the brain as an antioxidant has been widely discussed however, the dynamics of UA transport in the brain has remained unexplored. Further clarification of the regulatory mechanism of the level of UA in the central nervous system would be helpful for the realization of targeted neuroprotective therapy.

## Abbreviations

CSF: cerebrospinal fluid
KO: knockout
PD: Parkinson’s disease
PFA: paraformaldehyde
UA: uric acid
WT: wild-type
